# Intermittent fasting increases adult hippocampal neurogenesis

**DOI:** 10.1002/brb3.1444

**Published:** 2019-12-05

**Authors:** Sang‐Ha Baik, Vismitha Rajeev, David Yang‐Wei Fann, Dong‐Gyu Jo, Thiruma V. Arumugam

**Affiliations:** ^1^ Department of Physiology Yong Loo Lin School of Medicine National University of Singapore Singapore City Singapore; ^2^ School of Pharmacy Sungkyunkwan University Suwon Korea; ^3^ Neurobiology Programme Life Sciences Institute National University of Singapore Singapore City Singapore

**Keywords:** brain‐derived neurotrophic factor, hippocampus, intermittent fasting, neurogenesis, Notch

## Abstract

**Introduction:**

Intermittent fasting (IF) has been suggested to have neuroprotective effects through the activation of multiple signaling pathways. Rodents fasted intermittently exhibit enhanced hippocampal neurogenesis and long‐term potentiation (LTP) at hippocampal synapses compared with sedentary animals fed an ad libitum (AL) diet. However, the underlying mechanisms have not been studied. In this study, we evaluated the mechanistic gap in understanding IF‐induced neurogenesis.

**Methods:**

We evaluated the impact of 3 months of IF (12, 16, and 24 hr of food deprivation on a daily basis) on hippocampal neurogenesis in C57BL/6NTac mice using immunoblot analysis.

**Results:**

Three‐month IF significantly increased activation of the Notch signaling pathway (Notch 1, NICD1, and HES5), neurotrophic factor BDNF, and downstream cellular transcription factor, cAMP response element‐binding protein (p‐CREB). The expression of postsynaptic marker, PSD95, and neuronal stem cell marker, Nestin, was also increased in the hippocampus in response to 3‐month IF.

**Conclusions:**

These findings suggest that IF may increase hippocampal neurogenesis involving the Notch 1 pathway.

## Graphical abstract

Intermittent fasting (IF) is a dietary protocol where energy restriction is induced by alternate periods of ad libitum feeding and fasting. The present study has sought to investigate the relationship between IF and hippocampal neurogenesis. Our findings suggest that IF may increase hippocampal neurogenesis involving the Notch 1 pathway.

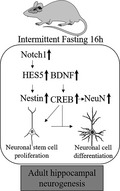

## 1 INTRODUCTION

Dietary restriction (DR) is defined as a decrease in energy consumption without reducing nutritional value. This simple dietary intervention has been shown in a wide range of experimental animals to extend lifespan and decrease the incidence of several age‐related diseases. The definition of DR has been expanded from an alternative description of caloric restriction (CR) to also encompass a broader scope of interventions, including short‐term starvation, periodic fasting, fasting‐mimetic diets, and intermittent fasting (IF; Mattson & Arumugam, 2018). IF has been proven to be advantageous to various organ systems in the body and acts as a mild metabolic stressor. It has been postulated that IF is able to cause powerful changes in the metabolic pathways in the brain via an increase in stress resistance, and breakdown of ketogenic amino acids and fatty acids (Bruce‐Keller, Umberger, McFall, & Mattson, 1999; Kim et al., 2018). Experimental studies have also shown that IF is neuroprotective against acute brain injuries such as stroke, and neurodegenerative diseases (Arumugam et al., 2010; Halagappa et al., 2007; Manzanero et al., 2014). In addition, recent studies have also shown that IF can lead to an increase in neurogenesis levels in the hippocampus (Manzanero et al., 2014).

In the adult brain, the niches of neuronal stem cells (NSCs) are located specifically at the subventricular zone (SVZ) of the lateral ventricles, and in the subgranular zone (SGZ) of the hippocampus. The ability of NSCs to maintain cerebral neurogenesis is controlled by the tight regulation of balanced events commencing from stem cell maintenance, to stem cell division and proliferation, to its differentiation into mature neurons, and finally their survival and functional integration into the brain parenchyma (Lathia, Mattson, & Cheng, 2008; Lledo, Alonso, & Grubb, 2006). The process of adult neurogenesis is highly regulated and is adaptable to environmental, morphological, and physiological cues, whereby cerebral performance is suited to function at optimal levels for a given environment. Studies have demonstrated that the proliferation of neural stem cells can be modified through metabolic perturbations experienced during high temperatures (Matsuzaki et al., 2009), physical activity (Niwa et al., 2016), and a high‐fat diet (Kokoeva, Yin, & Flier, 2005). Experimental studies from our group have also shown that IF increases neurogenesis in the hippocampus as a form of neuroprotection following acute brain injury such as ischemic stroke. Moreover, we established that the number of BrdU‐labeled cells in the dentate gyrus of IF mice was elevated (Manzanero et al., 2014). To measure cell proliferation without the confound availability of an exogenous marker BrdU, we established increases in the number of Ki67‐labeled cells in the dentate gyrus of mice on the IF diet, indicating enhancement of cell proliferation in these mice (Manzanero et al., 2014). In addition to our findings, previous work similarly demonstrated that using the every other day (EOD) IF regimen also increased BrdU‐labeled cell number in the hippocampus (Lee, Duan, & Mattson, 2002).

However, the molecular process involved in IF‐induced neurogenesis is not well understood. The Notch signaling pathway that is intricately involved in the determination of cell fate during brain development and adult neurogenesis may be a possible molecular process involved in IF‐induced neurogenesis (Lathia et al., 2008). In this study, we investigated the expression levels of molecular and cellular components of the hippocampal region, focusing specifically on Notch activation and associated proteins that are known to promote hippocampal neurogenesis such as brain‐derived neurotrophic factor (BDNF) and cAMP response element‐binding protein (CREB).

## 2 METHODS

### 2.1 Animals and experimental design

Male C57BL/6N mice were fed a normal chow diet comprised of a caloric basis of 58%, 24%, and 18% carbohydrate, protein, and fat, respectively, and were housed in the animal facilities at the National University of Singapore. During the entire experiment, the mice were housed in animal rooms at 20 to 22°C with 30%–40% relative humidity under a 12‐hr light/dark cycle. At 3 months of age, they were randomly assigned to ad libitum (AL), 12, 16, and 24‐hr diet groups. For 12 and 16‐hr groups, mice were fasted daily for either 12 hr (19:00–07:00) or 16 hr (15:00–07:00) for 3 months, whereas the 24‐hr group mice were fasted every other day for 3 months. The AL group was provided with food pellets ad libitum. The study was approved by the National University of Singapore Institutional Animal Care and Use Committee.

### 2.2 Immunoblot analysis

The hippocampus tissue was isolated from the entire brain and collected for immunoblot analysis. Detailed immunoblot analysis procedures were performed as previously described (Fann et al., 2013). Briefly, 15–30 ug of solubilized proteins was separated by electrophoresis in SDS‐PAGE, and the proteins transferred to a nitrocellulose membrane and blocked with 1% fish skin gelatin in 1 × TBS‐T at room temperature for 1 hr. The membranes were subsequently incubated at 4°C overnight with the following primary antibodies at a dilution of 1:1,000 for anti‐Notch 1 (Abcam, 52627; Cell Signaling, 3608S), anti‐BDNF (Abcam, 108319), anti‐NeuN (Abcam, 104225), anti‐Nestin (Merck Millipore, mab353), anti‐CREB (Cell Signaling, 9197S), anti‐p‐CREB (Cell Signaling, 9198S), anti‐Pin1 (Abcam, 76309), anti‐PS1 (Cell Signaling, 5643S), anti‐HES5 (Abcam, 194111), and anti‐PDS95 (Abcam, 18258). After washing within 1 × TBS‐T, the membranes were incubated with HRP‐conjugated secondary mouse or rabbit antibodies at room temperature for 1 hr.

### 2.3 Statistical analysis

All statistical analyses were carried out using GraphPad Prism software. Statistical significance was measured using one‐way ANOVA followed by Bonferroni's multiple comparison. All data are presented as mean ± *SD*.

## 3 RESULTS

### 3.1 Intermittent fasting increased markers for neurogenesis in the adult hippocampus

**Figure 1:**
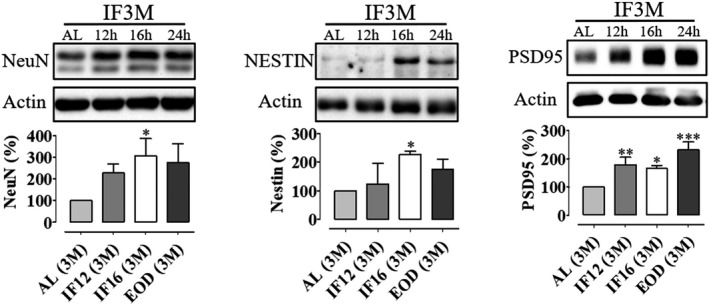
Intermittent fasting increases neurogenesis in the adult hippocampal brain. Representative immunoblots and quantification of NeuN, Nestin, and PSD95 in hippocampal lysates of mice subjected to daily intermittent fasting (IF12 hr, IF16 hr, and 24 hr [EOD]) for 3 months. Data are represented as mean ± *SD*. β‐Actin served as a loading control. *n* = 3 biological replicates were used in each experimental group. All IF groups were compared against ad libitum (AL) controls using one‐way ANOVA with Bonferroni post hoc test; *p < .05, **p < .01 and ***p < .001. EOD, every other day; IF, intermittent fasting; NeuN, neuronal nuclei; PSD95, postsynaptic density protein 95

Compared to AL mice, IF12, IF16, and EOD mice demonstrated significantly lower average body weight after 3 months of IF (Data not shown). However, there was no significant difference in average body weight between IF16 and EOD mice. Our data also indicated that there was no significant difference in the overall energy intake across mice subjected to IF in comparison with AL mice (Data not shown). The adult brain is comprised of self‐renewing multipotent neural stem cells that are accountable for neurogenesis and plasticity in precise regions of the brain. Nestin, a marker for neuronal stem cells, is expressed as immature forms of both neuronal epithelial cells and progenitor cell types in the brain. The present study demonstrated that intermittently fasted mice maintained for 3 months exhibited increased levels of NeuN in the hippocampus in comparison with the AL mice (Figure 1). Similarly, Nestin, a protein marker for precursor cells, followed the same trend as that of NeuN (Figure 1). These preliminary markers indeed show that there were increased levels of neurogenesis in the hippocampus in IF animals. In addition, we also found that there was a significant increase in the levels of PSD95 in the hippocampus of IF mice in comparison with AL mice (Figure 1). PSD95 is a major scaffolding protein in the excitatory postsynaptic density of neurons and is a potent regulator of synaptic strength. Elevated levels of PSD95 may indicate that IF not only leads to neurogenesis but also strengthens synaptic connections in the adult hippocampus. In order to further understand the molecular mechanisms that lead to IF‐mediated neurogenesis in the hippocampus, we next studied the expression and activation levels of the Notch 1 signaling pathway.

### 3.2 Intermittent fasting activates Notch signaling in the adult hippocampus

**Figure 2:**
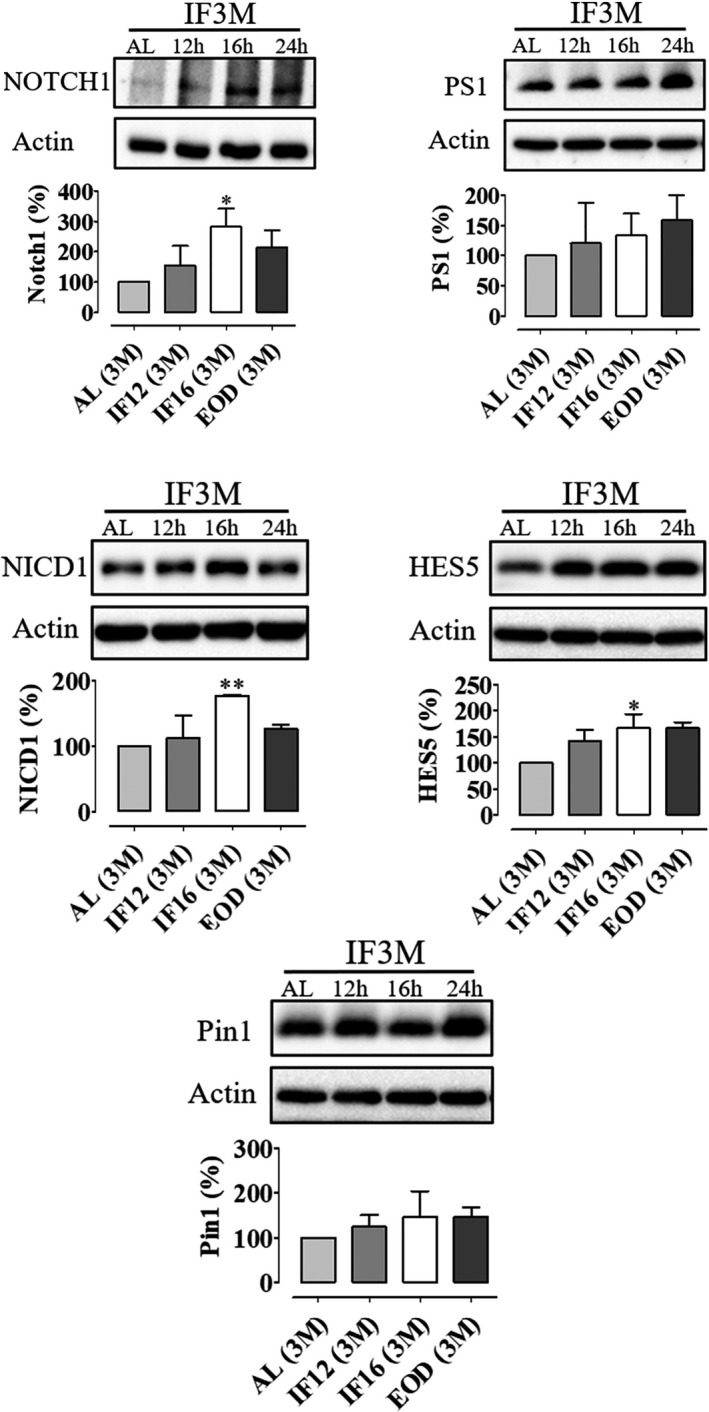
Intermittent fasting activates the Notch 1 signaling pathway in the adult hippocampal brain. Representative immunoblots and quantification of full‐ length Notch 1, NICD1, PS1, HES5, and Pin1 in hippocampal lysates of mice subjected to daily intermittent fasting (IF12 hr, IF16 hr, and 24 hr [EOD]) for 3 months. Data are represented as mean ± *SD*. β‐Actin served as a loading control. *n* = 3 biological replicates were used in each experimental group. All IF groups were compared against ad libitum (AL) controls using one‐way ANOVA with Bonferroni post hoc test; *p < .05 and **p < .01. EOD, every other day; IF, intermittent fasting; NICD1, notch intracellular domain‐1; Pin1, peptidyl‐prolyl cis‐trans isomerase NIMA‐interacting 1; PS1, presenilin 1

The Notch signaling pathway has long been established to be essential in the maintenance of neural stem cells in the mammalian brain (Hitoshi et al., 2002). Subsequent studies have established Notch 1 signaling to be essential in maintaining a reservoir of undifferentiated cells in order to ensure continuity of adult hippocampal neurogenesis (Ables et al., 2010). To test the hypothesis that IF may promote neurogenesis by activating the Notch signaling pathway, we investigated the main markers involved in the Notch 1 signaling pathway, especially the expression of full‐length Notch 1, Notch intracellular domain (NICD1), transcription factor HES5, and presenilin 1 (PS1). PS1 is known to have catalytic activity and is one of the subunits in the γ‐secretase complex that cleaves the transmembrane‐anchored cytoplasmic domain of Notch 1 to activate its downstream pathway (Arumugam et al., 2018; Cheng, Choi, Sobey, Arumugam, & Jo, 2015). Our data showed evidence that the Notch 1 signaling pathway was activated due to an upregulation of Notch 1, NICD1, and HES5 following IF (Figure 2). We also analyzed the expression level of Pin1 (Figure 2). Pin1 is a peptidyl‐isomerase that is well known to stabilize NICD1 by inhibiting ubiquitination (Baik et al., 2015). However, our data showed that IF did not alter the expression of Pin1. This may indicate that IF upregulates Notch 1 signaling independently of post‐translational modification.

### 3.3 Intermittent fasting increases the CREB/BDNF axis in the adult hippocampus

**Figure 3:**
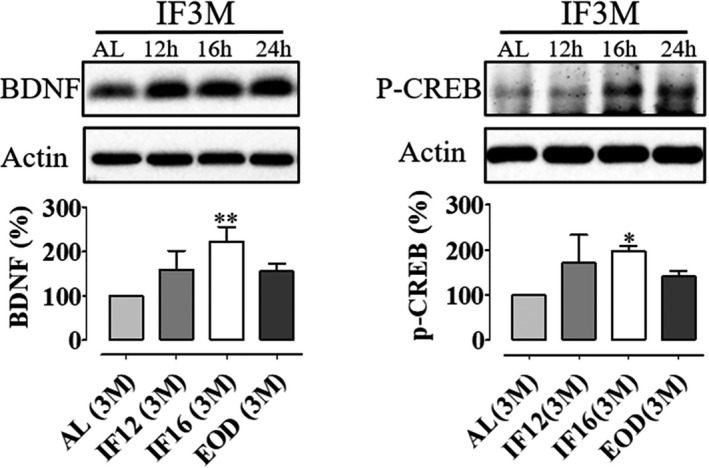
Intermittent fasting increases the BDNF/CREB signaling pathway in the adult hippocampal brain. Representative immunoblots and quantification of BDNF and p‐CREB in hippocampal lysates of mice subjected to daily intermittent fasting (IF12 hr, IF16 hr, and 24 hr [EOD]) for 3 months. Data are represented as mean ± *SD*. β‐Actin served as a loading control. *n* = 3 biological replicates were used in each experimental group. All IF groups were compared against ad libitum (AL) controls using one‐way ANOVA with Bonferroni post hoc test; *p < .05 and **p < .01. BDNF, brain‐derived neurotrophic factor; EOD, every other day; IF, intermittent fasting; p‐CREB, phosphorylated cAMP response element‐binding protein

Intermittent fasting leads to liver glycogen store depletion and lipolysis of free fatty acids (FFAs), which are then released into the blood. The FFAs are metabolized in the liver to generate the ketones, acetone, acetoacetate (AcAc), and β‐hydroxybutyrate (BHB) and transported into the brain (Mattson, Moehl, Ghena, Schmaedick, & Cheng, 2018). This metabolic switching confers the brain to a neuroprotective state against injury and diseases through the activation of the brain‐derived neurotrophic factor (BDNF) signaling pathway. At the presynaptic terminal, BDNF is released into the intracellular space in response to the activation of glutamate receptors. This may be a stress response mechanism toward the fluctuation in energy metabolism during IF. BDNF is also involved in the downstream activation of transcription factors that are involved in neurogenesis and energy balance, including cAMP response element‐binding protein (CREB). The transcription of BDNF is in turn controlled by CREB, hence establishing a positive feedback loop between BDNF and CREB. In addition, BDNF allows an increase in transcription of PSD95 to the cell surface membrane via the Trk pathway (Mattson et al., 2018; Yoshii & Constantine‐Paton, 2010). It was established that the Notch signaling pathway collaborates with the BDNF and CREB signaling pathways to allow differentiation of stem cells into mature neurons (Brai et al., 2015; He et al., 2019; Schölzke & Schwaninger, 2007). It was also previously established that CR and IF increase BDNF levels to protect against acute and chronic brain injury (Arumugam et al., 2010; Maswood et al., 2004). To test whether BDNF/CREB levels increased following IF conditions, we analyzed the expression levels of these two proteins. Our data showed that the expression of both BDNF and p‐CREB was upregulated in the hippocampus of IF animals in comparison with AL animals (Figure 3).

## 4 DISCUSSION

Prophylactic IF has been shown to promote longevity as well as ameliorate the development and manifestation of age‐related diseases such as cardiovascular, neurodegenerative, and metabolic diseases in many animal studies. It has also been postulated that IF is able to cause changes in the metabolic pathways in the brain, which leads to stress resistance capacity of brain cells (Kim et al., 2018). It was shown that protein chaperones (HSP‐70 and GRP‐78) and neurotrophic factors (BDNF and FGF2) were increased in brain cells in response to IF (Arumugam et al., 2010). In addition, IF was shown to increase hippocampal neurogenesis (Manzanero et al., 2014). The present study was to elucidate the impact of IF on key proteins that that regulates neurogenesis in the hippocampus.

**Figure 4:**
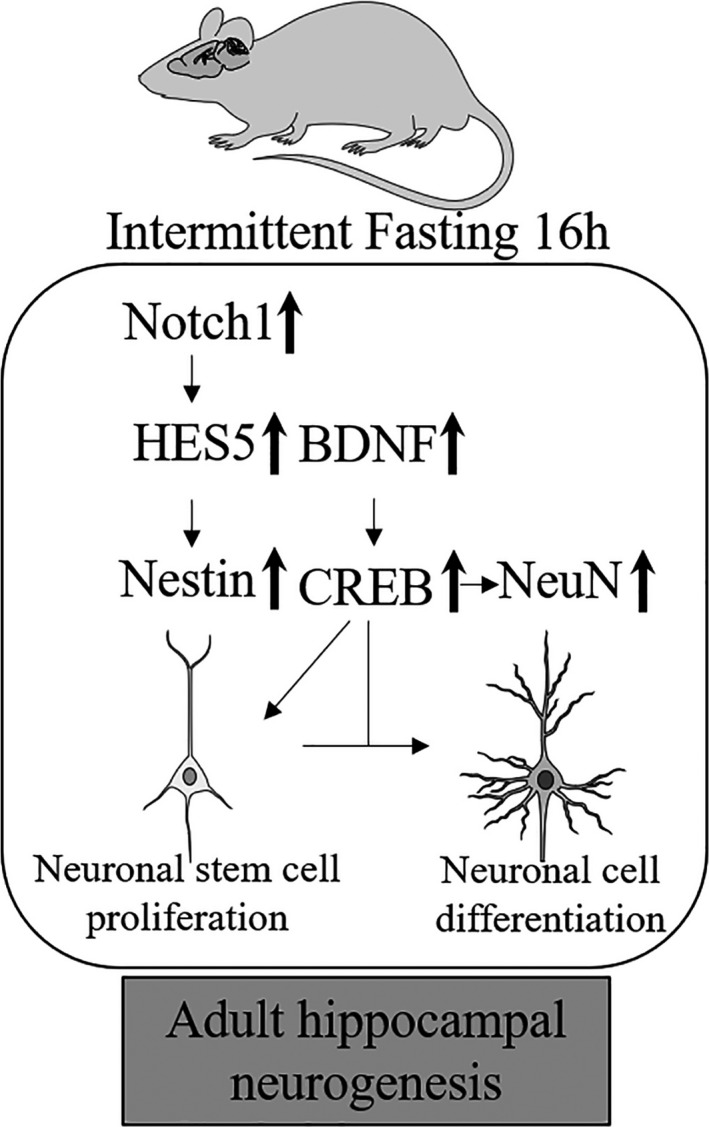
Schematic diagram of intermittent fasting‐mediated pathways that induce adult hippocampal neurogenesis. Intermittent fasting was shown to activate the Notch 1 signaling pathway, which caused the transcription factor, HES5, to induce an upregulation of Nestin, a marker of neuronal stem cells that led to increased neurogenesis. In addition, intermittent fasting was shown to activate the CREB signaling pathway, which caused an upregulation of neurotrophic factor, BDNF, that led to increased levels of Nestin and neurogenesis. BDNF, brain‐derived neurotrophic factor; CREB, cAMP response element‐binding protein; NeuN, neuronal nuclei

In mammals, NSCs are located in the SVZ and SGZ niches within the hippocampus, and are the basis for adult neurogenesis. These cells are either in their dormant, active, or quiescent state. This makes the neurogenesis capacity highly regulated and controlled within the region (Yamashima, Tonchev, & Yukie, 2007). In the adult mouse brain, the Notch family are the gatekeeper for adult neurogenesis, specifically the interplay between Notch 1 and Notch 2 (Imayoshi & Kageyama, 2011). The maintenance of adult neurogenesis is a delicate mechanism that is highly dependent on proper external and internal biological stimuli, and hence raises an important question—whether these NSCs have any response to a hormesis mechanism such as IF through the Notch signaling pathway and how its mechanisms are demonstrated. Notch 1 is specifically involved in the regulation of postmitotic dendrite development in cortical neurons during adult neurogenesis (Ding, Gao, Ding, Fan, & Chen, 2016). In the dentate gyrus region, Notch 1 is expressed extensively and is important in synaptic plasticity of neurons. This was confirmed by studies that showed downregulation of Notch 1 in the hippocampus reduced synaptic plasticity of neurons in the brain (Brai et al., 2015). Moreover, Notch 1 deletion was shown to reduce the number of neural stem cells in the brain (Shih & Holland, 2006). Downstream of Notch signaling is HES5, one of NICD's target genes. HES5 plays an important role during mammalian development, where it is involved in the formation of neurospheres (Ohtsuka, Sakamoto, Guillemot, & Kageyama, 2001). Following neurodevelopment, in the adult stage, HES5 acts as a transcription factor that moves to the nucleus when activated, to increase the transcription of many target genes, including Nestin (Venkatesh et al., 2017). Nestin is often involved in the proliferation of NSC population in the hippocampal region. Our study demonstrates that Notch signaling and BDNF/CREB pathways may play a crucial role in adult hippocampal neurogenesis induced by IF, particularly by promoting Nestin expression, which is essential for stem cell proliferation (Figure 4).

Our study has also provided an analysis of IF‐induced neurogenesis following different IF regimen, allowing a better understanding of IF regulation on the hippocampus. While all three IF regimens showed an increase in proteins that regulate hippocampal neurogenesis, only IF16 caused a significant increase in most of these protein expression. We have previously established that different IF regimen may induce differential metabolic switching processes, which may result in varying degrees of metabolic adaptation (Kim et al., 2018; Ng et al., 2019) and different effects on hippocampal neurogenesis. In conclusion, our findings suggest that IF may increase hippocampal neurogenesis involving the Notch 1 pathway.

## CONFLICT OF INTEREST

The authors declare no conflict of interest.

## Data Availability

The data that support the findings of this study are available from the corresponding author upon reasonable request.
